# An organometallic hybrid antibiotic of metronidazole with a Gold(I) N-Heterocyclic Carbene overcomes metronidazole resistance in *Clostridioides difficile*

**DOI:** 10.1007/s00775-024-02064-y

**Published:** 2024-06-26

**Authors:** Rolf Büssing, Arne Bublitz, Bianka Karge, Mark Brönstrup, Till Strowig, Ingo Ott

**Affiliations:** 1 Institute of Medicinal and Pharmaceutical Chemistry, Technische Universität Braunschweig, Beethovenstr. 55, 38106 Braunschweig, Germany; 2 Department of Microbial Immune Regulation, Helmholtz Centre for Infection Research GmbH, Inhoffenstrasse 7, 38124 Braunschweig, Germany; 3 Department of Chemical Biology, Helmholtz Centre for Infection Research GmbH, Inhoffenstrasse 7, 38124 Braunschweig, Germany

**Keywords:** Antibacterial, Bioorganometallics, Carbene, Gold, Metronidazole

## Abstract

Antimicrobial resistance (AMR) has been emerging as a major global health threat and calls for the development of novel drug candidates. Metal complexes have been demonstrating high efficiency as antibacterial agents that differ substantially from the established types of antibiotics in their chemical structures and their mechanism of action. One strategy to exploit this potential is the design of metal-based hybrid organometallics that consist of an established antibiotic and a metal-based warhead that contributes an additional mechanism of action different from that of the parent antibiotic. In this communication, we describe the organometallic hybrid antibiotic **2c**, in which the drug metronidazole is connected to a gold(I) N-heterocyclic carbene warhead that inhibits bacterial thioredoxin reductase (TrxR). Metronidazole can be used for the treatment with the obligatory anaerobic pathogen *Clostridioides difficile* (*C. difficile*), however, resistance to the drug hampers its clinical success. The gold organometallic conjugate **2c** was an efficient inhibitor of TrxR and it was inactive or showed only minor effects against eucaryotic cells and bacteria grown under aerobic conditions. In contrast, a strong antibacterial effect was observed against both metronidazole-sensitive and -resistant strains of *C. difficile*. This report presents a proof-of-concept that the design of metal-based hybrid antibiotics can be a viable approach to efficiently tackle AMR.

**Graphical abstract:**

A metronidazole-gold hybrid metalloantibiotic with high efficacy against resistant C. difficile 
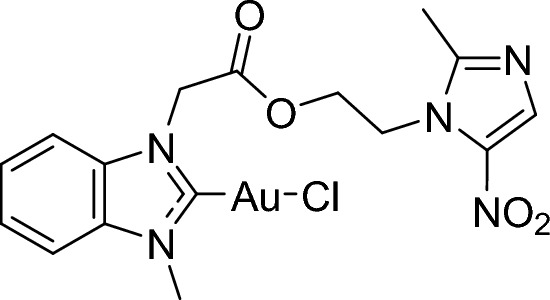

**Supplementary Information:**

The online version contains supplementary material available at 10.1007/s00775-024-02064-y.

## Introduction

Antimicrobial resistance (AMR) has become a global health problem with a sharply increasing relevance in the treatment of bacterial infections [1]. Metal-based drugs might play a key role in fighting AMR, as they differ significantly from the existing types of antibiotics and could in consequence be less susceptible to resistance of microbial pathogens. A systematic survey of the antibacterial activity of various types of metals implies their exceptional, yet hardly leveraged potential for antibiotic drug discovery [2–4].

Among the many investigated metal-based antibacterial agents, gold complexes have been attracting increasing attention recently [5–21]. Gold complexes are among the strongest inhibitors of the enzyme thioredoxin reductase, which is involved in the cellular redox regulation of both eukaryotic and many prokaryotic cells. In particular many Gram-positive bacteria, which lack a glutathione/glutathione reductase system, are highly dependent on a functional TrxR. For the gold-containing therapeutic auranofin it has been convincingly demonstrated that this mechanism is connected to its antibacterial effects and the drug, approved for other indications, has been considered for repurposing as an antibacterial agent [6, 19, 22]. The inhibition of bacterial TrxR has been confirmed for an increasing number of different gold compounds, suggesting that the inhibition of this enzyme could be a key player for the antibacterial effects of gold compounds in general [8, 11, 20, 21, 23]. Because auranofin and many other gold complexes trigger cytotoxic effects in eukaryotic (i.e. human) cells, their potential as safe antibiotics might be limited.

**Fig. 1:**
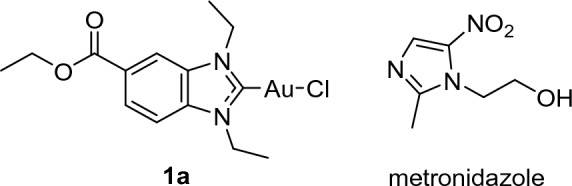
Structures of the anti-infective gold NHC complex **1a** [20] and metronidazole

Recently, we have reported on organometallic gold(I/III) complexes with *N*-heterocyclic carbene (NHC) ligands, many of which are efficient inhibitors of bacterial TrxR (see Fig. 1a for an example) [8, 20, 21, 24, 25]. Importantly, in agreement with the proposed mechanism of action based on TrxR inhibition, the complexes were highly active against Gram-positive bacteria, but poorly active or inactive against Gram-negative bacteria. However, the proliferation inhibition in human cancer and Gram-positive bacterial cells remained in a comparable concentration range.

One strategy to overcome toxicity limitations and AMR at the same time, is the design of metal-based hybrid antibiotics [26–29]. Such conjugates consist of an established antibacterial drug and a metal partial structure, which triggers antibacterial effects by a mode of action that is different from that of the selected antibiotic [2]. Here we selected metronidazole (Fig. 1) for the design of gold organometallic hybrid antibiotics, which is an antibiotic of the nitroimidazole class that inhibits nucleic acid synthesis by forming nitroso radicals. Metronidazole is activated by (partial) reduction that occurs usually in anaerobic bacteria, whereas the drug has little activity against aerobic bacteria and human cells. Metronidazole is, for example, applied in the therapy of infections with the obligate anaerobic bacterium *Clostridioides difficile* (*C. difficile*), which causes severe gastrointestinal infections. However, treatment success is diminished by the occurrence of metronidazole resistance. Based on the Gram-positive nature of *C. difficile*, we hypothesized that a TrxR-inhibiting gold organometallic conjugate with metronidazole could restore the activity against metronidazole-resistant *C. difficile*. In this report we provide the proof-of-concept for this approach.

## Results

**Scheme 1:**
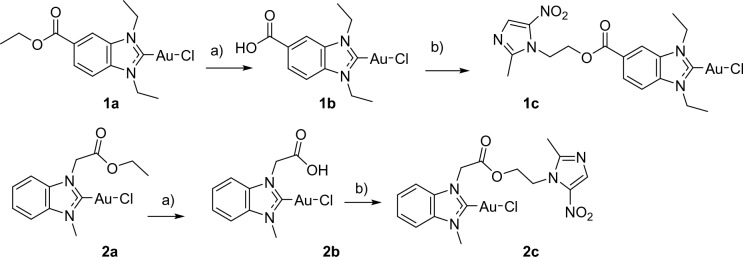
Synthesis of compounds **1c** and **2c**. **a** NaOH **b** EDC/DMAP

The hydroxy group of metronidazole offers an ideal position for forming conjugates without changing the pharmacophore. Regarding the gold NHC moiety, we selected complex **1a**, for which we had observed antibacterial activity and TrxR inhibition recently [20], as a starting point for attaching metronidazole by a two-step ester hydrolysis/re-esterification at the benzimidazole backbone of the NHC (see scheme 1). Similarly, complex **2a** was designed to introduce metronidazole at the side chain of the NHC ligand. Accordingly, the free carboxylic acids **1b** [30] and **2b** were obtained from **1** and **2a** by reaction with NaOH. The target hybrid organometallics **1c** and **2c** were prepared by Steglich-like esterification with the hydroxy group of metronidazole. This coupling strategy had been previously successfully applied by Gibson et al. for other metal-based drug candidates, i.e. platinum(IV) complexes [31, 32].

The compounds were characterized by ^1^H- and ^13^C-NMR and mass spectroscopy, which were fully consistent with the proposed structures. The high purity of all compounds was confirmed by elemental analyses that differed less than 0.5% from the theoretical values.

The two conjugates **1c** and **2c** differ in their solubility in organic solvents. While **2c** was soluble in most organic solvents and could be purified by column chromatography with dichloromethane and methanol as eluents, **1c** showed limited solubility in organic solvents that prevented chromatographic purification. Such different properties might be the consequence of the more rigid nature of the ester moiety of **1c** at the aromatic ring system, in comparison to the more flexible ester moiety of **2c**.

The ability of the organometallic hybrid antibiotics **1c** and **2c** to inhibit bacterial TrxR was evaluated in an assay using the purified enzyme from *E. coli* in comparison to the gold complexes auranofin and the free acids **1b** and **2b**. In general, all gold complexes were very efficient inhibitors of TrxR with IC_50_ values in the range of 0.15–0.36 µM (Table 1). The most active inhibitors were the metronidazole conjugates **1c** and **2c** with IC_50_ values that were approximately half of those of the respective free acid derivatives, **1b** and **2b**, and slightly surpassed the activity of the reference drug auranofin.

**Table 1:** Inhibition of bacterial TrxR from *E. coli*

	IC_50_ (µM)
Auranofin	0.210 ± 0.030
**1b**	0.297 ± 0.031
**2b**	0.364 ± 0.103
**1c**	0.145 ± 0.038
**2c**	0.175 ± 0.017

Low cytotoxicity against mammalian cells is of advantage when developing novel antibiotics. Here, the antiproliferative effects were examined in human A-549 lung cancer, HT-29 colon carcinoma, MDA-MB-321 breast cancer and Vero E6 non-tumorigenic epithelial kidney cells of the African green monkey (Table 2). Metronidazole was inactive as expected (all IC_50_ values > 100 µM). The free acid derivatives, **1b** and **2b**, as well as the metronidazole conjugates, **1c** and **2c**, were also largely inactive (all IC_50_ values > 60 µM) except for minor antiproliferative effects of **2c** in MDA-MB-231 cells (IC_50_ = 31.8 µM). Complex **2a**, which is the ethyl ester derivative of **2c**, was included in the study and showed minor to moderate cytotoxicity (IC_50_ values in the range of 9–26 µM, 9.6 µM against MDA-MB-231) that was in all cases 3–4 times stronger than that of the metronidazole conjugate **2c**. Such a strong reduction of the antiproliferative effects upon coupling of the gold carbene moiety to metronidazole is highly promising regarding a possible safe application of the complexes as antibiotics.

**Table 2:** Antiproliferative effects (IC_50_ values in µM ± standard deviation, *n* = 2–4) against several human tumorigenic and non-tumorigenic mammalian cell lines

	A-549	HT-29	MDA-MB-231	Vero E6
Auranofin	4.2 ± 0.6	10.5 ± 4.8	1.2 ± 0.3	2.6 ± 0.1
Metronidazole	> 100	> 100	> 100	> 100
**2a**	26.1 ± 3.7	19.9 ± 5.1	9.6 ± 2.7	25.8 ± 4.6
**1b**	> 75	66.3 ± 8.8	74.6 ± 1.3	> 100
**2b**	> 100	> 80	65.7 ± 8.2	> 100
**1c**	> 100	> 100	> 100	> 100
**2c**	81.9 ± 2.8	80.7 ± 5.9	31.8 ± 4.1	> 95

**Table 3:** MIC values [μM] ± standard error (*n* =  ≥ 3); MRSA = methicillin-resistant *S. aureus*

	MRSA	*E. faecium*	*E. coli*	*P.aeruginosa*	*A. baumannii*	*K. pneumoniae*
Metronidazole	> 350	> 350	> 350	> 350	> 350	> 350
Auranofin	< 0.2	0.6 ± 0.2	> 30	> 30	7.8 ± 7.1	> 30
**1b**	142.0 ± 0.0	142.0 ± 0.0	142.0 ± 0.0	> 142.0	> 142.0	> 142.0
**2b**	151.4 ± 0.0	> 151.4	151.4 ± 0.0	> 151.4	> 151.4	> 151.4
**1c**	106.0 ± 0.0	44.2 ± 15.3	> 106.0	> 106.0	> 106.0	> 106.0
**2c**	37.1 ± 16.0	43.3 ± 16.0	111.2 ± 71.7	> 111.2	> 111.2	> 111.2

The antibacterial effects were determined in a panel of aerobic Gram-positive (methicillin-resistant S*. aureus* MRSA, *Enterococcus faecium*) and Gram-negative (*Escherichia coli, Pseudomonas aeruginosa, Acinetobacter baumannii, Klebsiella pneumoniae*) pathogenic bacteria strains as minimal inhibitory concentration (MIC values) (Table 3). Metronidazole was not active under the aerobic conditions as expected. The gold reference compound auranofin showed strong activity against the Gram-positive stains and was lower active or inactive against the Gram-negative strains, in line with previous reports [6, 20, 25]. The free acid derivatives **1b** and **2b** were inactive (MIC values > 100 µM) against all bacteria strains. Except for **1c** in MRSA, the metronidazole conjugates **1c** and **2c** showed only moderate activity (MIC values: 37–44 µM) against Gram-positive strains and were also inactive against all the Gram-negative bacteria. The preference for Gram-positive bacteria is in good agreement with our recent reports on various gold NHC complexes [6, 20, 25].

**Fig. 2:**
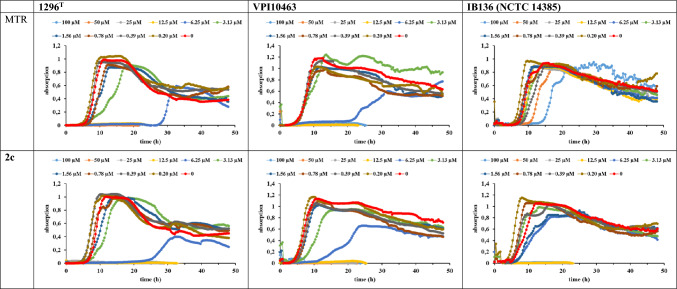
Antibacterial effects of metronidazole (MTR) and **2c** in dosages from 0.2 to 100 µM against different strains of *C. difficile*

Next, we evaluated the antibacterial activity of **2c** in concentrations up to 100 µM against two metronidazole-sensitive (1296^ T^, VPI10463) and one metronidazole-resistant IB136 (NCTC 14385)[33] strain of *C. difficile* in comparison to metronidazole under anaerobic conditions (Fig. 2). Preliminary studies with **1c** had been hampered by solubility issues under the assays conditions and therefore it was excluded from further experiments. Metronidazole displayed the expected strong activity against the sensitive strains at concentrations of 12.5 µM and above, leading to a complete block of bacterial growth without recovery, whereas no dosage of the drug could terminate the proliferation of the resistant strain. Complex **2c** blocked bacterial growth at the same concentration in all strains, including the metronidazole-resistant strain IB136 (NCTC14385), clearly confirming its ability to overcome metronidazole resistance in *C. difficile*. In addition, the activity against the highly toxin-producing strain VPI10463 is of particular interest, as toxins are causative for the pathogenicity of *C. difficile*. At a concentration of 6.25 µM both metronidazole and **2c** were effective inhibitors of the sensitive bacteria, however, with a recovery of growth after extended exposure. At the same concentration against the resistant *C. difficile* strain IB136, **2c** also showed strong effects with recovery of bacterial proliferation, whereas metronidazole remained completely inactive. To evaluate the importance of the metronidazole partial structure of **2c**, complex **2b** without metronidazole partial structure was evaluated under identical conditions and remained inactive against all *C. difficile* strains (Figures S1–S3), suggesting that the intact hybrid antibiotic is required for triggering bioactivity.

## Conclusions

Conjugates **1c** and **2c**, which consist of a gold NHC structure linked to metronidazole, were prepared and characterized. The TrxR inhibitory activity of the gold NHC moiety was not only maintained in the two metronidazole conjugates, in fact **1c** and **2c** surpassed the activity of the respective free acid derivatives **1b** and **2b**. Both organometallic hybrid antibiotics **1c** and **2c** were non-toxic or showed low effects against human cancer cell lines, a mammalian normal cell line, and several Gram-negative and Gram-positive bacteria. Complex **2c** overcame metronidazole resistance in *C. difficile* at low micromolar dosages. In conclusion, the design of hybrid gold organometallic antibiotics might provide an useful strategy to obtain novel resistance-breaking metal-based antibacterial drug candidates with a favorable toxicity profile.

## Experimental

### General

The reagents were purchased from Sigma-Aldrich, Alfa Aesar or TCI and used without additional purification steps. All reactions were performed without precautions to exclude air or moisture. ^1^H and ^13^C NMR were recorded on a DRX-400 AS, AVIIIHD 500 or AVII 600. A Finnigan LCQDeca or a Finnigan MAT 95 was used to record the ESI mass spectra. The elemental analyses were performed using a Flash EA 1112 (Thermo Quest CE Instruments). Absorption measurements for TrxR inhibition and antiproliferative activity were performed on a Perkin-Elmer 2030 Multilabel Reader VICTOR^TM^ X4. Compound **1a** [20] and 1-(ethoxycarbonylmethyl)-3-methylbenzimidazolium bromide [34] were prepared as described.

#### Chlorido-(1,3-diethyl-5-carboxybenzimidazol-2-ylidene)gold(I) (1b)

The compound was prepared according to a reported method with minor modifications [30]. Compound **1a** (158.0 mg, 0.33 mmol) was suspended in 18 mL absolute ethanol and a volume of 18 mL NaOH (42.3 mg, 1.06 mmol) in purified water was added under constant stirring. The mixture was heated for 65 min under reflux conditions and directly afterwards the volume was reduced using a rotary evaporator. The pH of the mixture was set to 1–2 by using diluted hydroxylic acid, extracted with dichloromethane, and the combined organic phases were dried over sodium sulphate. The volume was reduced using a rotary evaporator, the remaining solution was overlayed with n-hexane, and the precipitate formed over time was isolated, washed with n-pentane, and dried at 40 °C under reduced pressure. yield: 71.2 mg (0.158 mmol, 48%), ^1^H-NMR (400.4 MHz, DMSO-d_6_): δ [ppm] = 13.29 (s, 1H, COOH), 8.38 (d, J = 1.4 Hz, 1H, C(Ar)H), 8.06 (dd, J = 8.6 Hz, 1.4 Hz, 1H, C(Ar)H), 7.95 (d, J = 8.6 Hz, 1H, C(Ar)H), 4.62–4.51 (m, 4H, NCH_2_CH_3_), 1.46 (t, J = 7.2 Hz, 6H, NCH_2_CH_3_); ^13^C-NMR (100.7 MHz, DMSO-d^6^): δ [ppm] = 178.5 (1C, NCAuN), 166.8 (1C, COOH), 135.0 (1C, C(Ar)), 132.1 (1C, C(Ar)), 127.0 (1C, C(Ar)), 125.5 (1C, C(Ar)H), 113.5 (1C, C(Ar)H), 112.1 (1C, C(Ar)H), 43.6 (1C, NCH_2_CH_3_), 43.5 (1C, NCH_2_CH_3_), 15.5 (1C, NCH_2_CH_3_), 15.3 (1C, NCH_2_CH_3_).; MS (ESI) m/z = 437.4 [M-Cl–H^+^Na]^+^, 456.2 [M-Cl + MeCN]^+^; CHN(calc./found): C 31.98/32.02, H 3.13/3.13, N 6.22/6.01.

#### Chlorido-(1,3-diethyl-5-((2-(2-methyl-5-nitroimidazol-1-yl)ethoxy)carbonyl)benzimidazol-2-ylidene)gold(I) 1c

**1b** (111.6 mg, 0.248 mmol), 4-dimethylaminopyridine (10.6 mg, 0.087 mmol) and metronidazole (40.3 mg, 0.235 mmol) were suspended in 0.5 mL dimethylformamide and stirred for 10 min at room temperature. A solution of 1-ethyl-3-(3-dimethylaminopropyl)carbodiimide hydrochloride (EDC, 45.6 mg, 0.238 mmol) in 0.5 mL DMF was added, the mixture was stirred for 78 h, poured in distilled water, and stirred for 15 min. The formed precipitate was collected by filtration, washed with distilled water, and dried at 40 °C under reduced pressure. Yield: 79.5 mg (0.132 mmol, 53%); ^1^H-NMR (500.3 MHz, DMSO-*d6*): δ [ppm] = 8.24 (d, *J* = 1.4 Hz, 1H, *C*(Ar)H), 8.06 (s, 1H, *C*(Im)H), 7.99 (d, *J* = 8.6 Hz, 1H, *C*(Ar)H), 7.94 (dd, *J* = 8.6 Hz, 1.4 Hz, 1H, *C*(Ar)H), 4.83–4.75 (m, 2H, NC*H*_2_CH_2_O), 4.75–4.65 (m, 2H, NCH_2_C*H*_2_O), 4.60–4.51 (m, 4H, NC*H*_2_CH_3_), 2.49 (s, 3H, NCC*H*_3_N), 1.49–1.43 (m, 6H, NCH_2_C*H*_3_); ^13^C-NMR (125.8 MHz, DMSO-*d6*): δ [ppm] = 179.0 (1 C, N*C*AuN), 164.8 (1 C, *C*OO), 151.5 (1 C, N*C*CH_3_N), 138.6 (1 C, C(Im)NO_2_), 135.5 (1 C, C(Ar)), 133.2 (1 C, C(Ar)), 132.1 (1 C, C(Im)H), 125.2 (1 C, C(Ar)), 125.1 (1 C, C(Ar)H), 113.3 (1 C, C(Ar)H), 112.5 (1 C, C(Ar)H), 63.2 (1 C, NCH_2_*C*H_2_O), 44.6 (1 C, N*C*H_2_CH_2_O), 43.7 (1 C, N*C*H_2_CH_3_), 43.6 (1 C, N*C*H_2_CH_3_), 15.5 (1 C, NCH_2_*C*H_3_), 15.4 (1 C, NCH_2_*C*H_3_), 13.9 (1 C, NC*C*H_3_N); MS (ESI) m/z = 626.1 [M + Na]^+^, 939.3 [2 M-AuCl_2_]^+^, 1171.2 [2 M-Cl]^+^, 1229.2 [2 M + Na]^+^; CHN (calc./found) C 35.81/35.88, H 3.51/3.63, N 11.60/11.25.

#### Chlorido-(1-(2-ethoxy-2-oxoethyl)-3-methylbenzimidazol-2-ylidene)gold(I) 2a

1-(Ethoxycarbonylmethyl)-3-methylbenzimidazolium bromide (300.0 mg, 0.369 mmol) and silver oxide (51.2 mg, 0.221 mmol) in dichloromethane (20 mL) were stirred over night in the dark at room temperature. Chlorido(dimethylsulfide)gold(I) (119.6 mg, 0.406 mmol) was added, the mixture was stirred for another 40 h at room temperature, filtered using Celite®, the solvent was evaporated and the product was dried at 40 °C under reduced pressure. Yield: 413.4 mg (0.917 mmol, 91%); ^1^H-NMR (600.1 MHz, DMSO-*d*_*6*_): δ [ppm] = 7.84–7.76 (m, 2H, C(Ar)*H*), 7.55–7.46 (m, 2H, C(Ar)*H*), 5.43 (s, 2H, NC*H*_2_CO_2_), 4.21 (q, *J* = 7.1 Hz, 2H, OC*H*_2_CH_3_), 4.05 (s, 3H, NC*H*_3_), 1.24 (t, *J* = 7.1 Hz, 3H, OCH_2_C*H*_3_); ^13^C-NMR (150.9 MHz, DMSO-*d*_*6*_): δ [ppm] = 178.4 (1C, N*C*AuN), 167.4 (1C, *C*O_2_), 133.0 (1C, *C*(Ar)), 132.9 (1C, *C*(Ar)), 124.6 (1C, *C*(Ar)H), 124.4 (1C, *C*(Ar)H), 112.1 (1C, *C*(Ar)H), 112.0 (1C, *C*(Ar)H), 61.5 (1C, O*C*H_2_CH_3_), 49.0 (1C, N*C*H_2_CO_2_), 35.0 (1C, N*C*H_3_), 13.9 (1C, OCH_2_*C*H_3_); MS (ESI +) m/z = 473.0 [M + Na]^+^, 633.2 [2 M-AuCl_2_]^+^, 923.1 [2 M + Na]^+^; elemental analysis (calc./found) C 31.98/31.81, H 3.13/3.09, N 6.22/6.10.

#### Chlorido-(1-carboxymethyl-3-methyl)benzimidazol-2-ylidene)gold(I) 2b

Compound **2a** (242.6 mg, 0.538 mmol) was suspended in 20 mL absolute ethanol and a volume of 20 mL NaOH (72.3 mg, 1.808 mmol) in purified water was added under constant stirring. The mixture was heated for 65 min under reflux conditions and directly afterwards the volume was reduced using a rotary evaporator. The pH of the mixture was set to 2–3 by using diluted hydrochloric acid, extracted with dichloromethane, and the combined organic phases were dried over sodium sulphate. The volume was reduced to roughly 30–40% using a rotary evaporator, the remaining solution was overlayed with n-hexane, and the precipitate formed over time was isolated, washed with n-pentane, and dried at 40 °C under reduced pressure. Yield: 105.2 mg (0.249 mmol, 46%); ^1^H-NMR (500.3 MHz, DMSO-d_6_): δ [ppm] = 7.82–7.76 (m, 2H, C(Ar)H), 7.52–7.46 (m, 2H,C(Ar)H), 5.30 (s, 2H, NCH_2_), 4.04 (s, 3H, NCH_3_); ^13^C-NMR (125.8 MHz, DMSO-d_6_): δ [ppm] = 178.3 (1C, NCAuN), 168.6 (1C, COOH), 133.1 (1C,C(Ar)), 133.0 (1C, C(Ar)), 124.5 (1C, C(Ar)H), 124.3 (1C, C(Ar)H), 112.0 (2C, C(Ar)H), 49.2 (1C, NCH_2_), 35.0 (1C, NCH_3_); MS (ESI) m/z = 444.8 [M + Na]^+^, 808.7 [2 M-Cl]^+^, 830.7 [2 M + Na–H-Cl]^+^, 852.6 [2 M + 2Na-2H-Cl]^+^; CHN (calc./found) C 28.42/28.62, H 2.39/2.33, N 6.63/6.32.

#### Chlorido-(1-methyl-3-(2-(2-(2-methyl-5-nitroimidazol-1-yl)ethoxy)-2-oxoethyl)-benzimidazol-2-yliden)gold(I) 2c

**2b** (147.1 mg, 0.348 mmol), 1-ethyl-3-(3-dimethylaminopropyl)carbodiimide hydrochloride (EDC, 64.1 mg, 0.334 mmol), 4-dimethylaminopyridine (14.9 mg, 0.122 mmol) and metronidazole (56.6 mg, 0.331 mmol) were dissolved in 1.0 mL dimethlyformamide and stirred for 48 h at room temperature. The mixture was poured into 30 mL dichloromethane and washed with 25 mL of distilled water. The aqueous phase was extracted twice with 30 mL dichloromethane, the combined organic phases were dried over sodium sulphate, and the volume was reduced in a rotary evaporator to approximately 1 mL of a yellow solution. A volume of 3.0 mL dichloromethane was added, the solution was overlayed with n-hexane and left at − 20 °C. The precipitate was collected, washed with n-hexane, and purified by column chromatography over silica with dichloromethane/methanol (96/4) as eluent. Yield: 27.0 mg (0.047 mmol, 14%); ^1^H-NMR (500.3 MHz, DMSO-*d*_*6*_): δ [ppm] = 8.00 (s, 1H, NC*H*CNO_2_), 7.83 (dt, *J* = 8.1 Hz, 0.9 Hz, 1H, C(Ar)*H*), 7.71–7.65 (m, 1H, C(Ar)*H*), 7.57–7.45 (m, 2H, C(Ar)*H*), 5.42 (s, 2H, NC*H*_2_CO_2_), 4.58 (dd, *J* = 5.4 Hz, 4.2 Hz, 2H, OC*H*_2_CH_2_N), 4.52 (dd, *J* = 5.4 Hz, 4.1 Hz, 2H, OCH_2_C*H*_2_N), 4.05 (s, 3H, NC*H*_3_), 2.29 (s, 3H, NCC*H*_3_N); ^13^C-NMR (125.8 MHz, DMSO-*d*_*6*_): δ [ppm] = 178.4 (1C, N*C*AuN), 167.0 (1C, CH_2_*C*O_2_), 151.4 (1C, N*C*CH_3_N), 138.3 (1C, *C*NO_2_), 133.0 (2C, C(Met)H + C(Ar)), 132.7 (1C, C(Ar)), 124.6 (1C, C(Ar)H), 124.5 (1C, C(Ar)H), 112.2 (1C, C(Ar)H), 111.9 (1C, C(Ar)H), 63.9 (1C, NCH_2_*C*H_2_O), 48.9 (1C, N*C*H_2_CO_2_), 44.4 (1C, N*C*H_2_CH_2_O), 35.1 (1C, N*C*H_3_), 13.8 (1C, NC*C*H_3_N); MS (ESI +) m/z = 539.8 [M-Cl]^+^, 575.8 [M + H]^+^, 597.7 [M + Na]^+^; elemental analysis (calc./found) C 33.38/33.58, H 2.98/2.98, N 12.16/11.68.

#### Inhibition of bacterial TrxR (*E. coli*)

The TrxR (E.coli) inhibition assay was performed according to a previously published procedure [8]. It is partly based on the procedure developed by Lu et al.[35] and detects the formation of 5-TNB (5-thionitrobenzoic acid). Solutions of *E. coli* TrxR (35.4 U/mL) and of its substrate thioredoxin (Trx) *E. coli* (156 µg/mL) (both purchased from Sigma-Aldrich and diluted with distilled water) and fresh stock solutions of the test compounds (in DMF) were prepared (**1c** was administered as suspension). TE buffer (Tris–HCl 50 mM, EDTA 1 mM, pH 7.5) containing graded concentrations of the respective compounds (20 µL) or buffer without compounds (20 µL, as control) were mixed with TrxR solution (10 µL), Trx solution (10 µL), and a solution of NADPH (200 µM) in TE buffer (100 µL) in a well on a 96-well plate. As blank solution, 200 µM NADPH in TE buffer (100 µL) mixed with a DMF/buffer mixture (40 µL) was used (final concentrations of DMF: 0.5% v/v). The solutions on the 96-well plate were incubated for 75 min at 25 °C with moderate shaking. A volume of 100 µL of a reaction mixture (TE buffer containing 200 µM NADPH and 5 mM DTNB) was added to each well to initiate the reaction. After thorough mixing, the formation of 5-TNB was monitored by a microplate reader at 405 nm in 35 s intervals (10 measurements). The values were corrected by subtraction of the values for the blank solution. The increase in concentration of 5-TNB followed a linear trend (r^2^ ≥ 0.990) and the enzymatic activities were calculated as the gradients (increase in absorbance per second) thereof. Absence of interference with the assay components was confirmed by a negative control experiment for each test compound. The highest test compound concentration was used and the enzyme solution was replaced by TE buffer for this purpose. The IC_50_ values were calculated as the concentration of the compound decreasing the enzymatic activity of the positive control by 50% and are presented as the means and standard deviations of independent repeated experiments.

### Cell culture

A549 lung carcinoma, HT-29 colon carcinoma, MDA-MB-231 breast cancer, and Vero E6 monkey kidney cells were maintained in Dulbecco’s modified Eagle’s medium (DMEM; 4.5 g/L D-glucose, l-glutamine, pyruvate), supplemented with fetal bovine serum superior, standardized (Biochrom GmbH, Berlin, 10% v/v) and gentamycin (50 mg/L) with a weekly passage.

### Antiproliferative assay in tumorigenic and non-tumorgenic cells

The antiproliferative effects were determined according to a previously published procedure [20]. A volume of 100 µL of cell suspension of A549 cells, MDA-MB-231, HT-29 or Vero E6 cells was transferred into the wells of a 96-well plates and incubated at 37 °C under 5% CO_2_ for 72 h. Stock solutions of the compounds (**1b**, **1c**, and **2c** were suspended due to limited solubility) were freshly prepared in dimethylformamide (DMF) and diluted with the respective cell medium to obtain various concentrations (final concentration of DMF: 0.1% v/v). After 72 h (A549, HT-29, Vero E6) or 96 h (MDA-MB-231) of exposure, the biomass of the cells was determined via crystal violet staining and the IC_50_ value was determined as the concentration that caused 50% inhibition of cell proliferation relative to an untreated control. The results were calculated as the mean values of three independent experiments, unless stated otherwise.

#### Antibacterial effects against aerobic *bacteria*

The following strains were used and maintained at 37 °C in MHB (21 g/L Müller Hinton, pH 7.4) or TSY (30 g/L trypticase soy broth, 3 g/L yeast extract, pH 7.0–7.2) medium. *Acinetobacter baumannii* (DSM 30007, ATCC 19606) in MHB, *Escherichia coli* (DSM1116, ATCC 9637) in TSY, *Klebsiella pneumoniae* (DSM 11678, ATCC33495) in MHB, *Pseudomonas aeruginosa* PA7 (DSM 24068) in MHB, *Enterococcus faecium* (DSM 20477, ATCC 19434) in TSY, *Staphylococcus aureus* MRSA (DSM 11822, ICB 25701) in TSY). Minimum inhibitory concentration (MIC) values were determined following a standardized protocol in broth dilution assays. The compounds and auranofin were serially diluted (**1b** and **1c** showed limited solubility) starting from 64 µg/ml using a pipetting robot (epMotion, Eppendorf, Germany). Starting inocula of 2–8 × 105 colony-forming units per ml in MHB or TSY media at 37 °C were used, and serial dilutions were carried out in 384-well microtiter plates in duplicate. After incubation of the plates for 20 h at 37 °C, the absorbance at 600 nm was measured to determine the MIC value (Enspire Multimode Microplate Reader, Perkin Elmer Inc.). The MIC values for the tested compounds were determined in three independent experiments by a curve-fitting procedure using the GraphPad Prism software (Graphpad Software, Inc.).

#### Antibacterial effects against *C. difficile*

The *Clostridioides difficile* strains 1296^ T^ (DSM 1296), VPI10463 (ATCC 43255) and the metronidazole resistant strain IB136 (NCTC 14385)[33] (provided by Ulrich Nübel, DSMZ Braunschweig) were maintained in prereduced BHIS medium (37 g/L BHI, oxoid, 5% yeast extract, 1% L-cysteine) in a Coy-Laboratories anaerobic chamber (85% N_2_, 10% CO_2_, 5% H_2_) at 37 °C. Freshly prepared cultures were incubated until the exponential growth phase (OD_600_ = 0.2–0.4) and were adjusted to 1 × 10^6^ cfu/mL before inoculation. The test compounds were dissolved in DMSO and by two-fold dilution steps concentrations in the range from 200 µM to 0.39 µM in BIHS medium were obtained. The solutions (100µL) were added to 100 µL of a diluted *C. difficile* culture (total volume: 200 µL in BIHS, 5 × 10^5^ *C. difficile*, 100–0.195 µM test compound per well). The OD600-values over a period of 48 h were measured with a LogPhase 600 4-plate reader (BioTek) and compared with blank BHIS.

### Supplementary Information

Below is the link to the electronic supplementary material.

Supplementary file1 (PDF 920 KB)
