# Supplementary material for: An organometallic hybrid antibiotic of metronidazole with a Gold(I) N-Heterocyclic Carbene overcomes metronidazole resistance in Clostridioides difficile
Source: J Biol Inorg Chem. 2024 Jun 26;29(5):511–8. doi: 10.1007/s00775-024-02064-y (PMC11343806; doi:10.1007/s00775-024-02064-y)
Supplement: Supplementary file 1 — Supplementary file1 (PDF 920 KB) [file 775_2024_2064_MOESM1_ESM.pdf]

Supporting information

An Organometallic Hybrid Antibiotic of Metronidazole with a Gold(I) N-Heterocyclic Carbene Overcomes Metronidazole Resistance in *Clostridioides difficile*

Rolf Büssing [a], Arne Bublitz [b], Bianka Karge [c], Mark Brönstrup [c], Till Strowig [b], Ingo Ott [a]*

a) Institute of Medicinal and Pharmaceutical Chemistry, Technische Universität Braunschweig, Beethovenstr. 55, 38106 Braunschweig, Germany

b) Department of Microbial Immune Regulation, Helmholtz Centre for Infection Research GmbH, Inhoffenstrasse 7, 38124 Braunschweig, Germany

c) Department of Chemical Biology, Helmholtz Centre for Infection Research GmbH, Inhoffenstrasse 7, 38124 Braunschweig, Germany

TABLE OF CONTENTS

S2: Antibacterial effects of **2b** against different strains of *C. difficile* (Figures S1-S3)

S3: NMR spectra (Figures S4 – S13)

Antibacterial effects of 2b against different strains of C. *difficile*

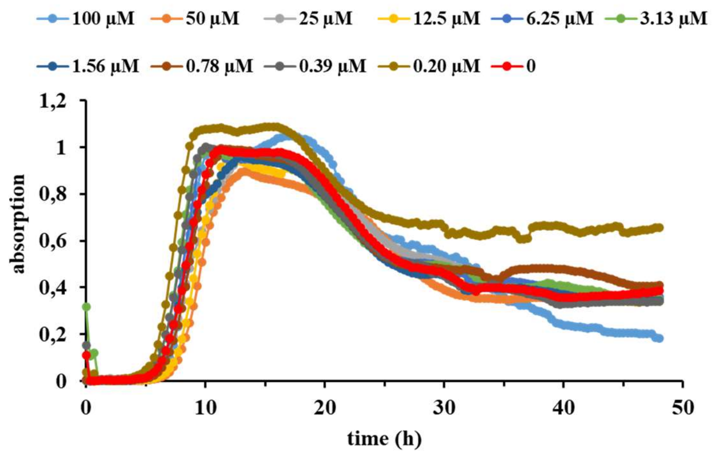

Figure S1: Antibacterial effects of **2b** in dosages from 0.2 to 100 µM against the *C. difficile* strain 1296^{T}

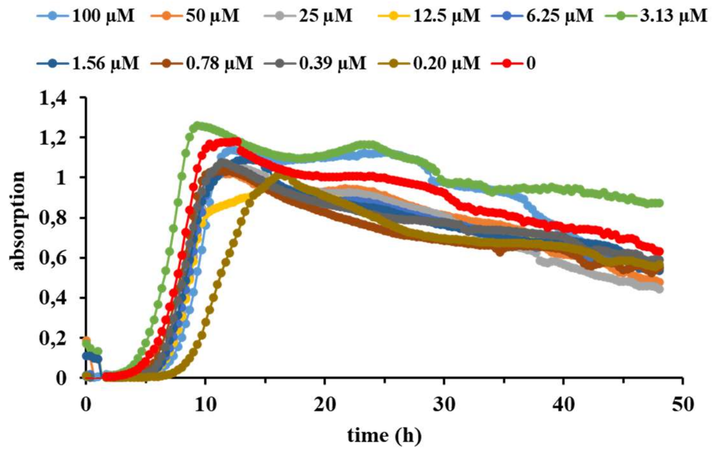

Figure S2: Antibacterial effects of **2b** in dosages from 0.2 to 100 µM against *the C. difficile* strain VPI10463

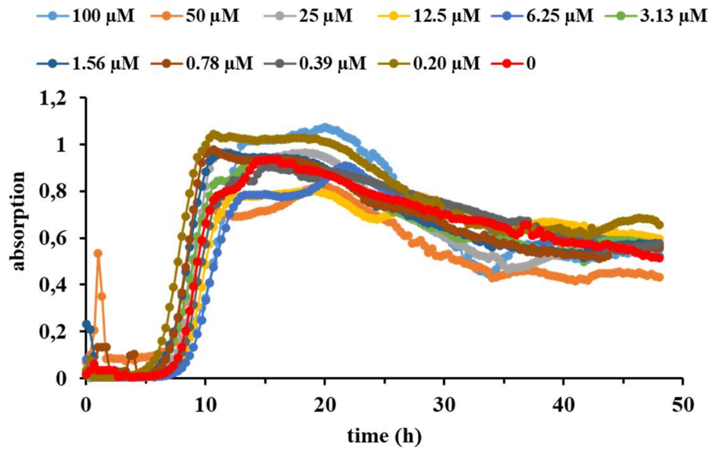

Figure S3: Antibacterial effects of **2b** in dosages from 0.2 to 100 µM against *the C. difficile* strain IB136 (NCTC 14385)

NMR Spectra

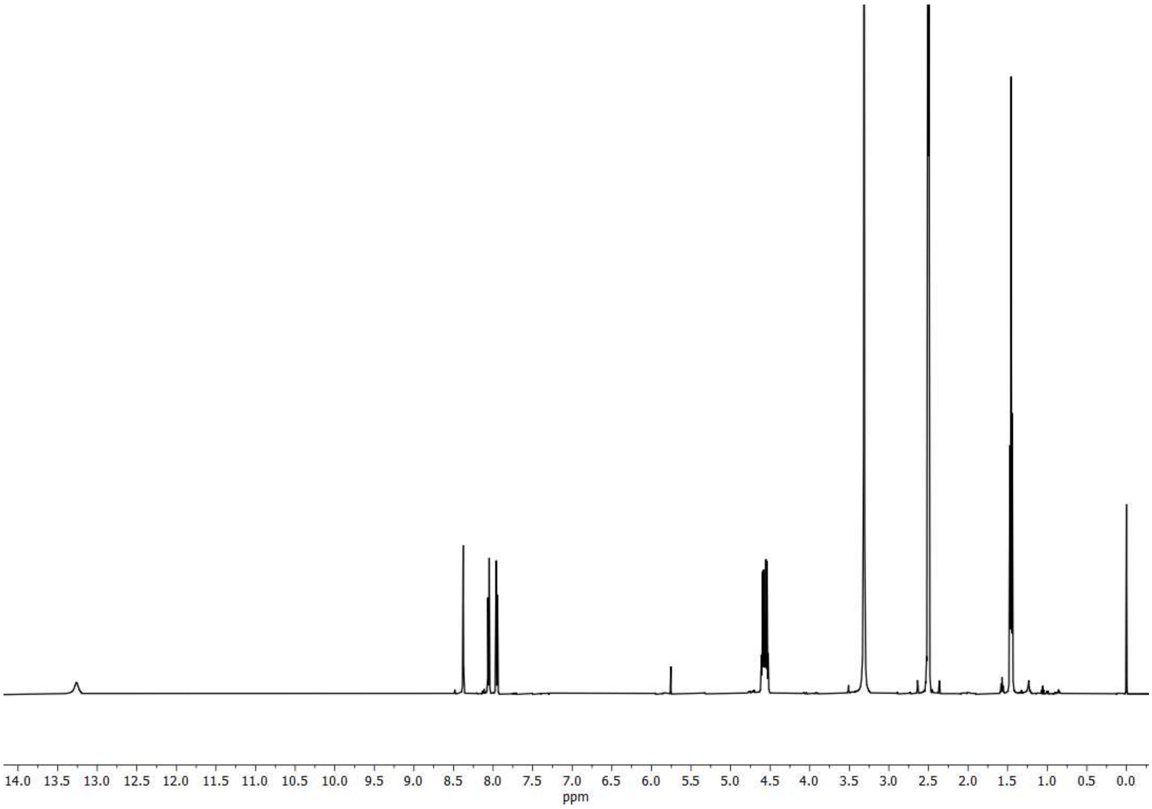

Figure S4: ^{1}H-NMR spectrum (400.4 MHz, DMSO-d_6) of **1b**

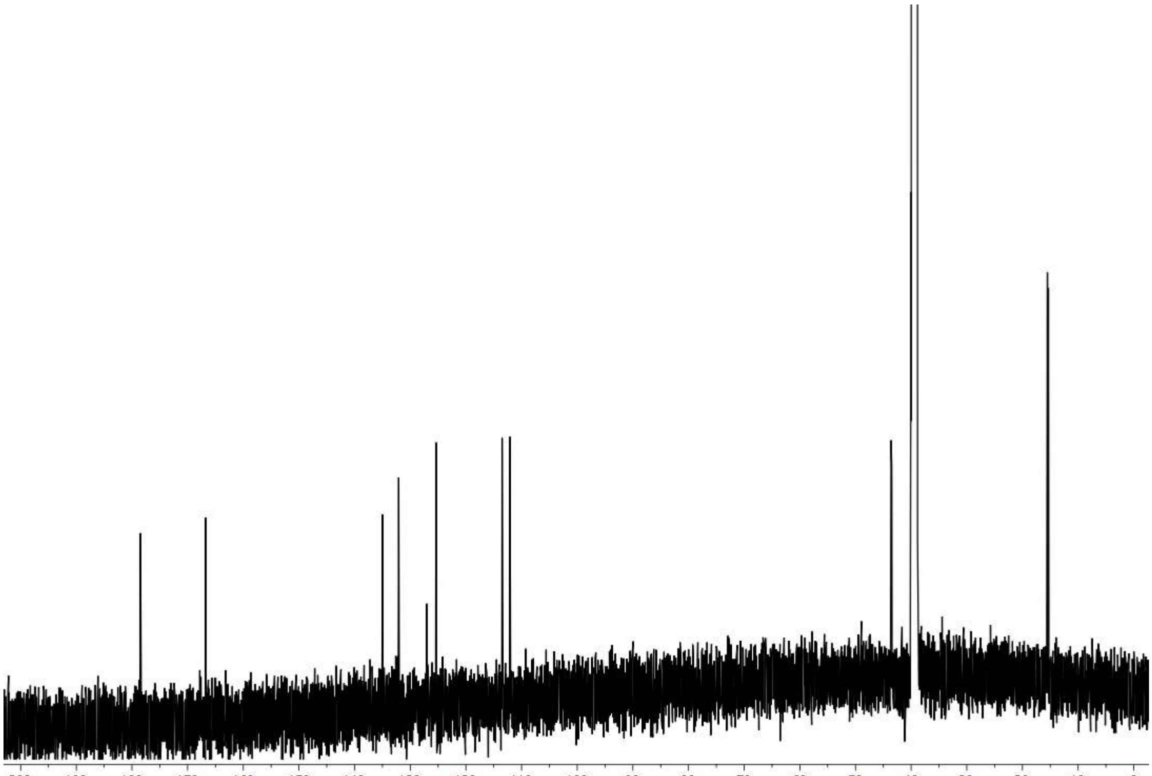

Figure S5: ^{13}C-NMR spectrum (100.7 MHz, DMSO-d_6) of **1b**

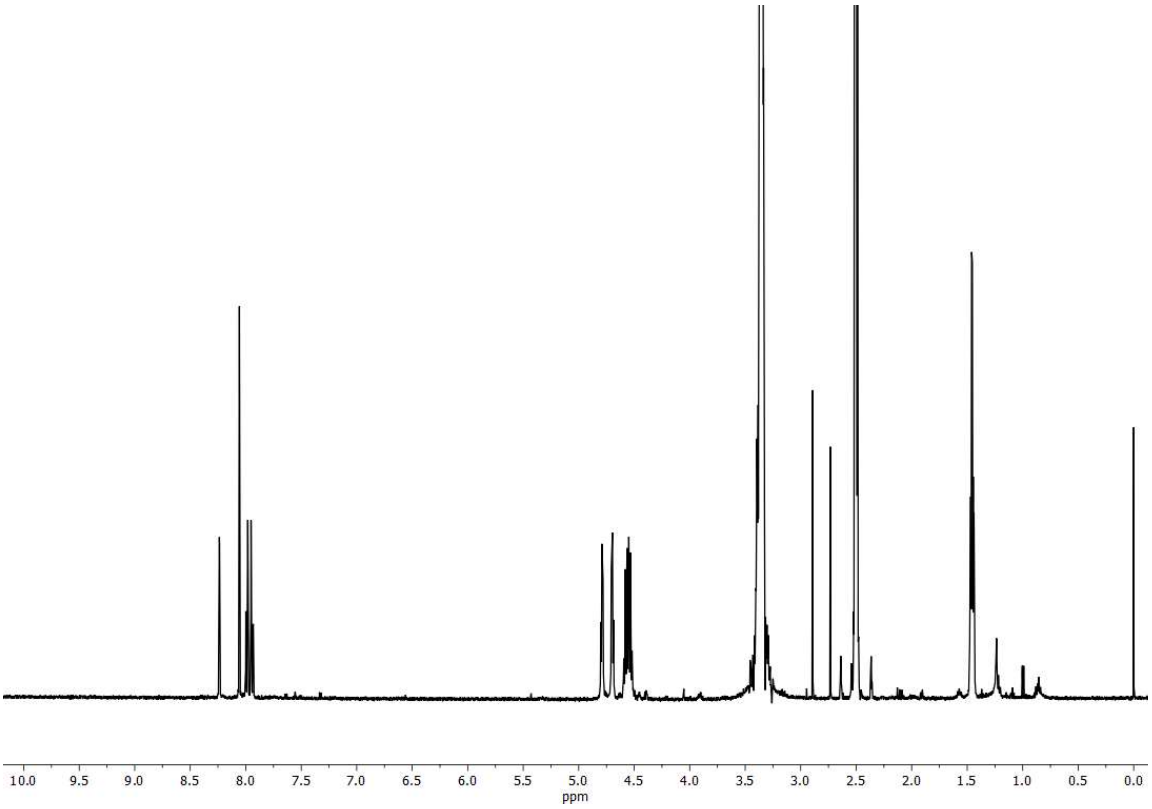

Figure S6: ^{1}H-NMR spectrum (500.3 MHz, DMSO-d_6) of **1c**

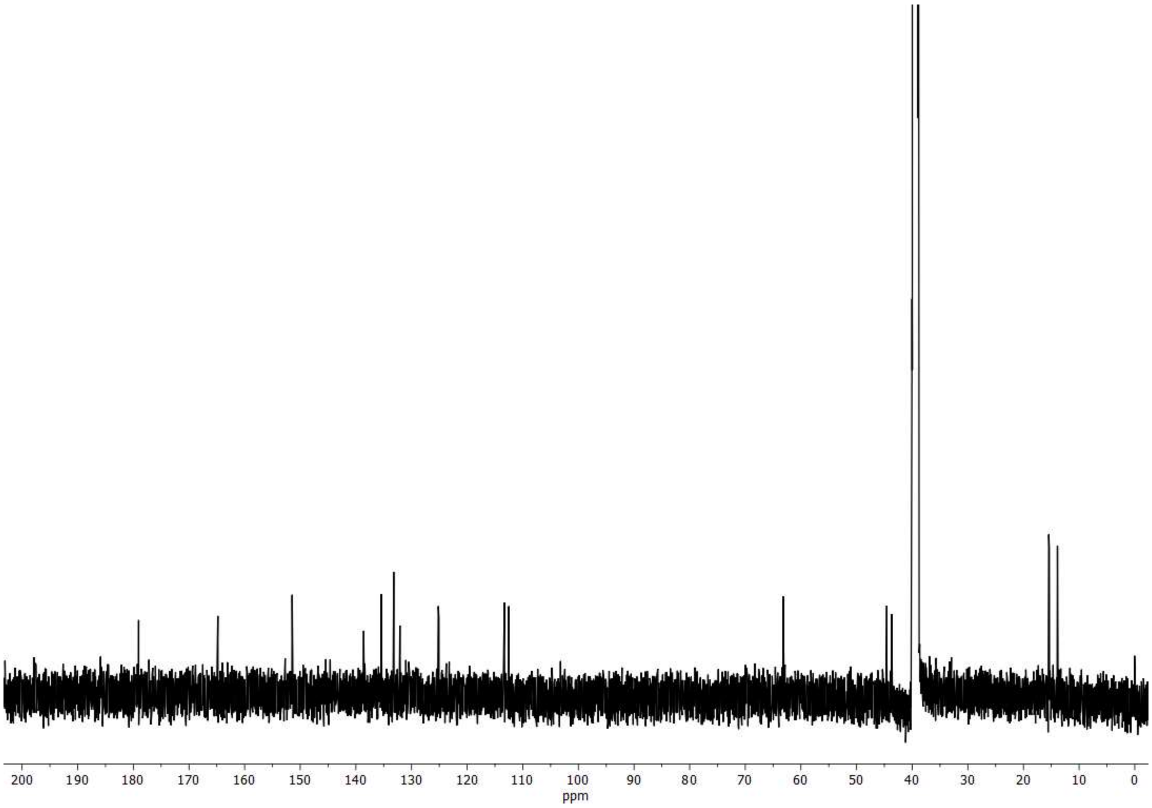

Figure S7: ^{13}C-NMR spectrum (125.8 MHz, DMSO-d_6) of **1c**

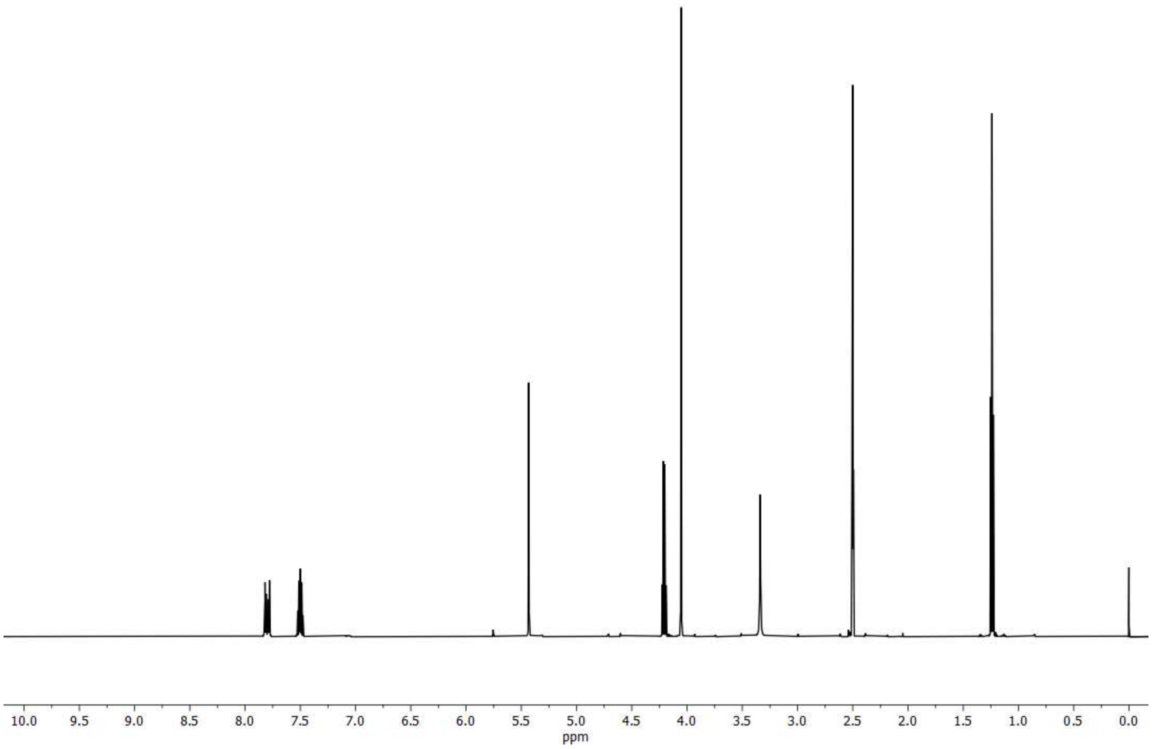

Figure S8: ^{1}H-NMR spectrum (600.1 MHz, DMSO-d_6) of **2a**

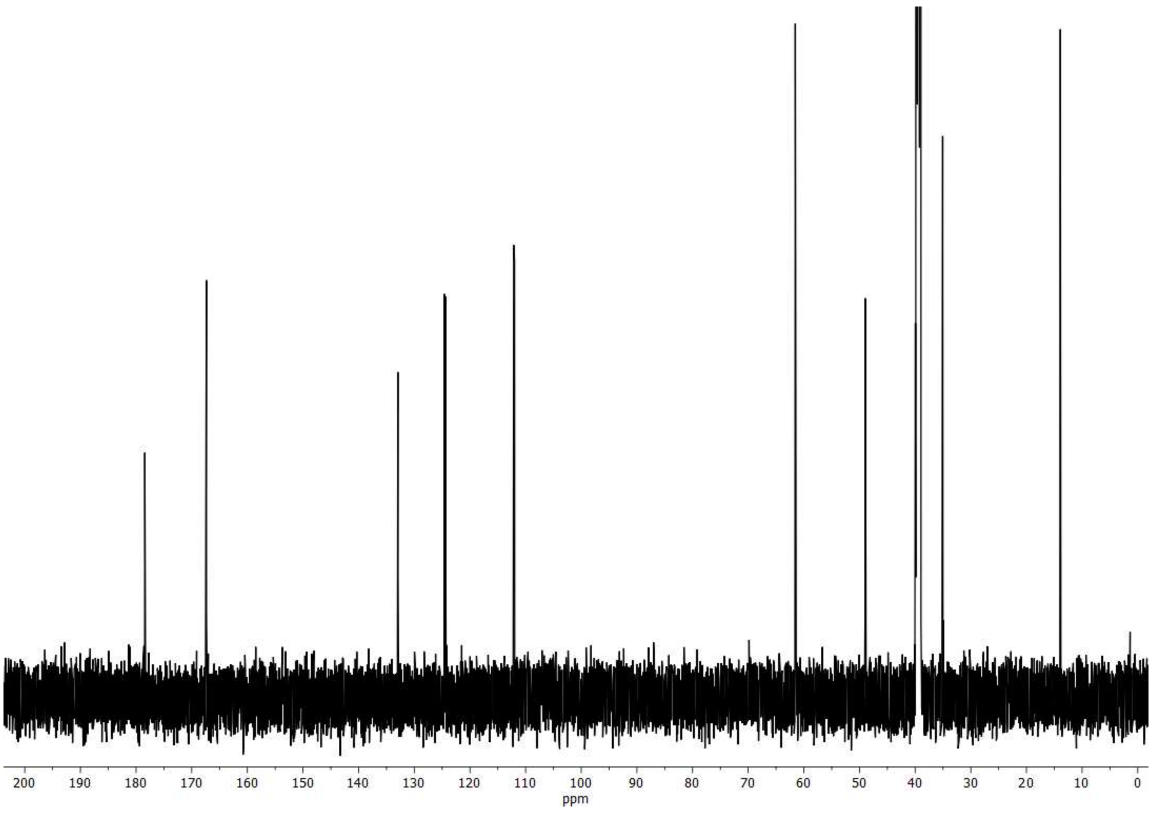

Figure S9: ^{13}C-NMR spectrum (150.9 MHz, DMSO-d_6) of **2a**

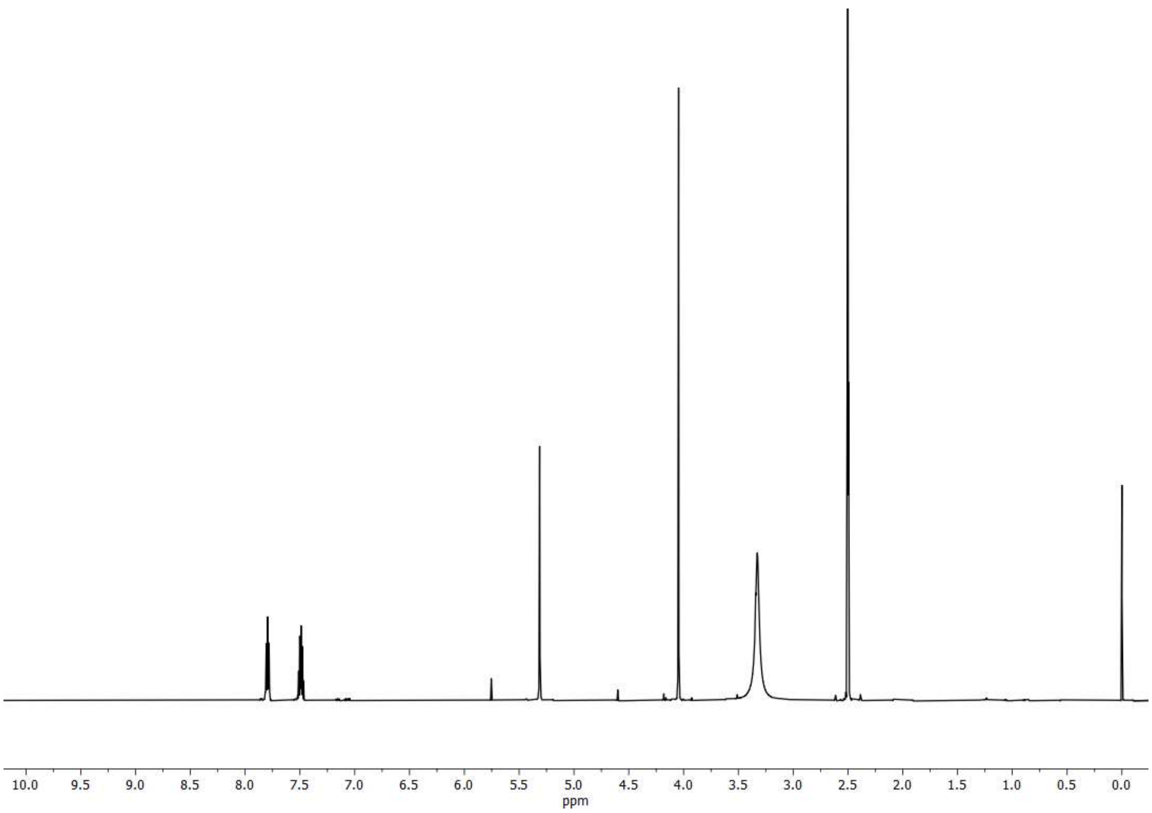

Figure S10: ^{1}H-NMR spectrum (500.3 MHz, DMSO-d_6) of **2b**

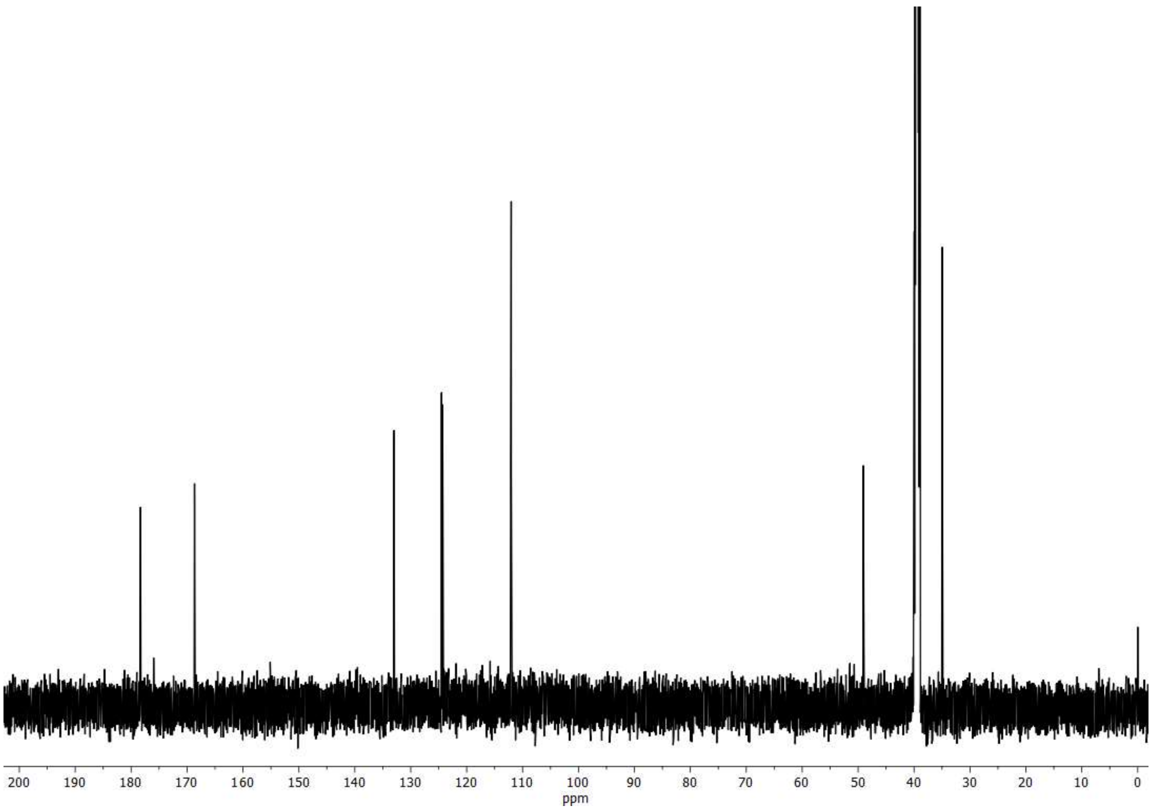

Figure S11: ^{13}C-NMR spectrum(125.8 MHz, DMSO-d_6) of **2b**

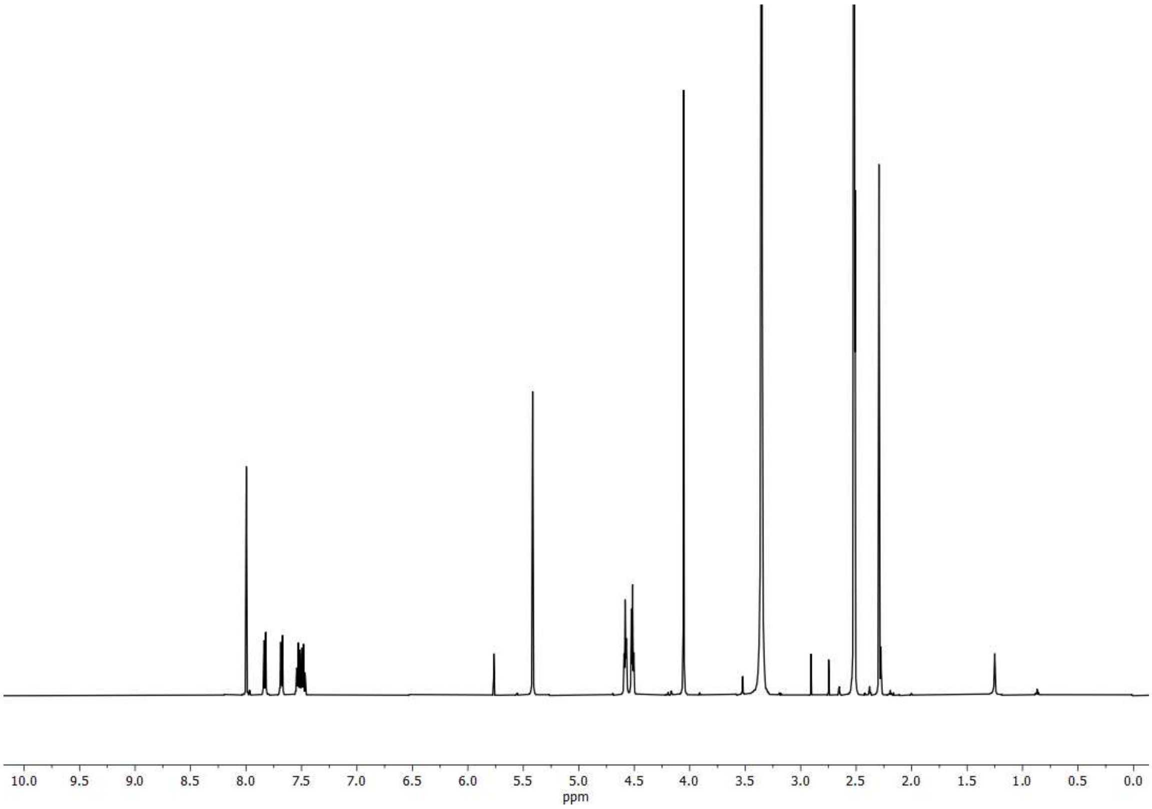

Figure S12: ^{1}H-NMR spectrum (500.3 MHz, DMSO-d_6) of **2c**

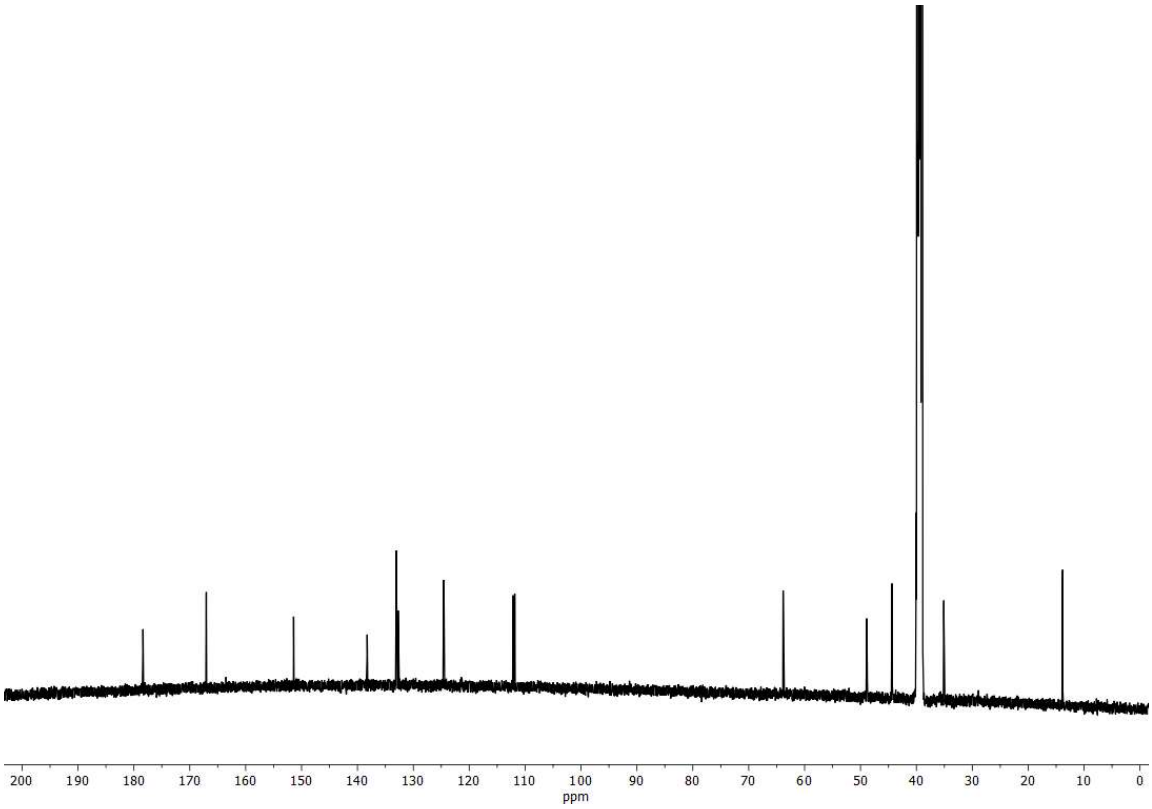

Figure S13: ^{13}C-NMR spectrum (125.8 MHz, DMSO-d$_6$) of **2c**
